# Alterations of the Gut Microbiome in Patients With Pituitary Adenoma

**DOI:** 10.3389/pore.2022.1610402

**Published:** 2022-08-04

**Authors:** Jinxian Hu, Jihu Yang, Lei Chen, Xiangbao Meng, Xiejun Zhang, Weiping Li, Zongyang Li, Guodong Huang

**Affiliations:** Department of Neurosurgery, Shenzhen Key Laboratory of Neurosurgery, Inst Translat Med, The First Affiliated Hospital of Shenzhen University, Shenzhen Second People’s Hospital, Shenzhen, China

**Keywords:** pituitary adenoma, invasive pituitary adenoma, gut microbiome, metagenome sequencing, noninvasive pituitary adenoma

## Abstract

Pituitary adenoma (PA) includes invasive pituitary adenoma (IPA) and noninvasive pituitary adenoma (NIPA), which are associated with the endocrine system. The gut microbiome plays an important role in human metabolism, but the association between the gut microbiome and pituitary adenoma remains unclear. A total of 44 subjects were enrolled in this study. Of these, 29 PA patients were further divided into IPA patients (*n* = 13) and NIPA patients (*n* = 16), while 15 healthy age-matched subjects were defined as control subjects. We collected faecal samples and characterized the gut microbial profiles by metagenomic sequencing using the Illumina X-ten platform. PLS-DA showed different microbial clusters among the three groups, and slightly different microbial ecological networks were observed. LEfSe analysis revealed significant alterations in the microbial community among PA patients. In particular, the enrichment of *Clostridium* innocuum, along with the reduced abundance of Oscillibacter sp. 57_20 and *Fusobacterium* mortiferum, were observed both in the IPA and NIPA groups compared to the control group. Moreover, PA patients could be effectively classified based on these bacteria using a support vector machine algorithm. In summary, this study demonstrated significant differences in the gut microbiome between PA patients and healthy controls. Future mechanistic experiments are needed to determine whether such alterations are a cause or consequence of pituitary adenoma.

## Introduction

As one of the most common primary brain tumours, pituitary adenoma (PA) occurs in ∼16% of intracranial tumour cases worldwide [1, 2]. Pituitary adenoma is clinically defined as invasive pituitary adenoma (IPA) or noninvasive pituitary adenoma (NIPA), and approximately 35% of pituitary adenomas are IPA [3], which exhibit invasive behaviours and actively invade surrounding tissues [3, 4]. PA can cause serious morbidity because of dysregulated pituitary hormone secretions, which are responsible for vital bodily functions, such as growth, blood pressure, reproduction, and metabolism [5]. A better understanding of the aetiology of PA is crucial for the improvement of clinical management and the development of new treatment options.

Gut microbiome from patients with schizophrenia has a lower alpha-diversity index and some disturbances of gut microbial composition than healthy control using 16S rRNA sequencing, which can modulate neurologic function through glutamate-glutamine-GABA cycle with fecal microbial transplantation study [6]. And a growing number of preclinical and clinical studies, including antibiotic use, fecal microbial transplantation, germ-free animal model and probiotic administration, detail the interactions between the gut microbiome and the central nervous system, improving our understanding the relationship between brain and gut [7, 8]. In addition, reduced bacterial diversity, altered representation of bacterial genes and metabolic pathways are observed in some endocrine system-related diseases, such as obesity, diabetes [9, 10]. These studies indicate that gut microbiome can modulate neurobehavioral traits, and endocrine functions [11]. Further, gut microbiome not only influence the brain function, brain behaviour, and neuroendocrine responses to stress, but its community and gastrointestinal permeability also be affected by the neuroendocrine system through hypothalamic–pituitary–adrenal (HPA) axis, points to a bidirectional communication between gut microbiome and neuroendocrine system [12]. Notably, a recent study reported that the gut microbiome in patients with newly diagnosed acromegaly has significantly lower bacterial diversity, changed Firmicutes/Bacteroidetes ratio and some genus using 16S rRNA sequencing, compared to healthy control [13]. These evidences indicate the potential association between gut microbiota and the occurrence and development of pituitary adenoma.

In this study, we aimed to add new evidence to the association between PA and the gut microbiome. By performing metagenomic sequencing, the distinction in the gut microbiome between healthy controls and PA patients was investigated.

## Materials and Methods

### Patient Cohort

Approval was obtained from the ethics committee of Shenzhen Second People’s Hospital [No. 20210617001]. The procedures used in this study adhere to the tenets of the Declaration of Helsinki. Informed consent was obtained from all individual participants included in the study. This was a cross-sectional study, and all participants were recruited from Shenzhen Second People’s Hospital from April to November 2020. After excluding those with complications of the endocrine system (such as diabetes, hyperthyroidism, acromegaly, etc.) and a family history of endocrine neoplasia, 44 subjects who agreed to provide faecal samples were recruited for our study. Written informed consent was obtained from all participants. In total, 29 diagnosed patients were categorized into the PA group, including 13 IPA patients and 16 NIPA patients, while 15 healthy subjects were categorized into the normal control (HC) group. All samples were confirmed by pathological examination. The diagnostic criteria for IPA included Knosp classification, intraoperative findings of tumour invasion, and Ki-67 labelling index according to previous studies [2, 14]. The demographic and clinical characteristics were derived from the electronic medical records of the hospital information system, including age, gender and body mass index, etc. Stool samples were collected from the first defecation (almost in the morning) using sterile swabs about 5 g within 3 days. The samples were snap-frozen in dry ice right after collection, and transferred to −80°C freezers in 30 min and stored with sterile tubes until subsequent experiments. To avoid the patient identification, we gave each study subject and sample with a unique subject ID and sample ID, respectively.

### DNA Extraction and Sequencing

Shotgun metagenome sequencing was performed based on the method of a previous study [15]. Briefly, total faecal genomic DNA was extracted using the Stool Genomic DNA Kit (CW2092S; CWBIO) according to the manufacturer’s instructions.

DNA concentrations were measured using a NanoDrop 2000 system (Thermo Fisher Scientific), and the DNA molecular size was estimated by agarose gel electrophoresis. The DNA library was constructed using the TruSeq DNA Sample Preparation Kit (Illumina, San Diego, CA, United States). Libraries were sequenced using the 150 base pair paired-end strategy on the Illumina X-ten platform at Novogene Bioinformatics Technology Co., Ltd. A total of 274.5 GB original sequencing data were obtained from 44 samples (6.2G per sample, ranged from 4.8 to 8.7 G) and deposited in the Sequence Read Archive (PRJNA799832), where 6.1 ± 0.41 G for IPA group, 6.5 ± 0.66 G for NIPA group, 6.0 ± 1.13 G for HC group.

### Bioinformatics and Statistical Analysis

Sequence adapter contamination and low-quality reads were discarded from the raw sequencing reads using Fastp software [16], and the remaining reads were filtered to eliminate human host DNA based on the human genome reference (hg19) using BWA software [17]. The relative abundance profiles of microbes were calculated using MetaPhlAn3 software with default parameters [18], and the abundance of microbial metabolic pathways against the MetaCyc database was calculated using HUMAnN3 software [18].

Alpha diversity was estimated on the basis of the microbial profile of each sample using Vegan’s diversity function in the R package. Partial least squares discriminant analysis (PLS-DA) was used to reveal taxonomic changes among the three groups, and variable importance in projection (VIP) scores were used to rank the ability of different taxa to discriminate between different groups. LEfSe (Linear Discriminate Analysis of Effect size) software was used to estimate the differentially abundant taxa between two groups with *p* < 0.05 [19]. The significantly different functional metabolic pathways were investigated using DESeq2 software [20]. The microbial ecological network was constructed using SpiecEasi software at the genus level and visualized with Gephi software [21].

Differences in characteristics were assessed with *t* tests (or analysis of variance, ANOVA) and chi-squared tests for continuous and categorical variables, respectively. All these analyses were performed using R software (v 3.6.3) at 0.05 significance levels.

A classification model was built to classify subjects with PA based on microbial profiles using the support vector machine (SVM) algorithm with 10-fold cross validation. The AUC (area under the curve) was calculated to evaluate the model performance.

## Results

### Characteristics of Study Participants

A total number of 44 participants were enrolled in this study. We only collected the subjects without complications of the endocrine system and a family history of endocrine neoplasia. According to the severity, the PA patients were further divided into IPA (*n* = 13) and NIPA (*n* = 16) subgroups. On the other hand, 15 healthy subjects were enrolled as the control group (HC). The demographic and clinical characteristics of all the participants are summarized in Table 1. They are all Han Chinese and live in Guangdong Province. There was no significant difference among the three groups in terms of age, BMI (body mass index) or sex. In addition, adrenocorticotropic hormone (ACTH), prolactin (PRL), thyroid stimulating hormone (TSH), luteinizing hormone (LH), follicle stimulating hormone (FSH), and growth hormone (GH) presented without significant differences between IPA and NIPA groups.

**TABLE 1:** Characteristics of the participants with pituitary adenoma and healthy controls.

	IPA group (*n* = 13)	NIPA group (*n* = 16)	HC group (*n* = 15)	*p*-value
Age (years)	40.31 ± 11.45	36.80 ± 8.34	42.47 ± 9.16	0.27
BMI (kg/m^2^)	23.76 ± 3.72	23.12 ± 3.01	22.04 ± 3.83	0.43
Male/Female	9/4	7/9	8/7	0.38
ACTH (pg/ml)	44.76 ± 34.86	39.87 ± 19.95	NA	0.65
PRL (ng/ml)	45.54 ± 69.04	29.20 ± 28.76	NA	0.40
TSH (mIU/L)	1.94 ± 0.96	1.46 ± 0.99	NA	0.20
LH (mIU/L)	2.45 ± 2.09	5.20 ± 777	NA	0.21
FSH (mIU/L)	6.33 ± 5.33	8.69 ± 13.78	NA	0.58
LH/FSH (ratio)	0.42 ± 0.32	0.67 ± 0.95	NA	0.40
GH (ng/ml)	4.14 ± 10.10	5.02 ± 9.85	NA	0.82

### Overall Structural Diversity of Gut Bacterial Communities

To evaluate the overall characteristics of the gut microbiota in the HC, NIPA and IPA groups, we compared the α-diversity estimators (Shannon index). No differences existed in the mean values of the Shannon index between IPA (2.40 ± 0.82 versus 2.29 ± 0.73, *p* = 0.73) and NIPA (2.65 ± 0.44 versus 2.29 ± 0.73, *p* = 0.11) groups, compared to the HC group.

**FIGURE 1:**
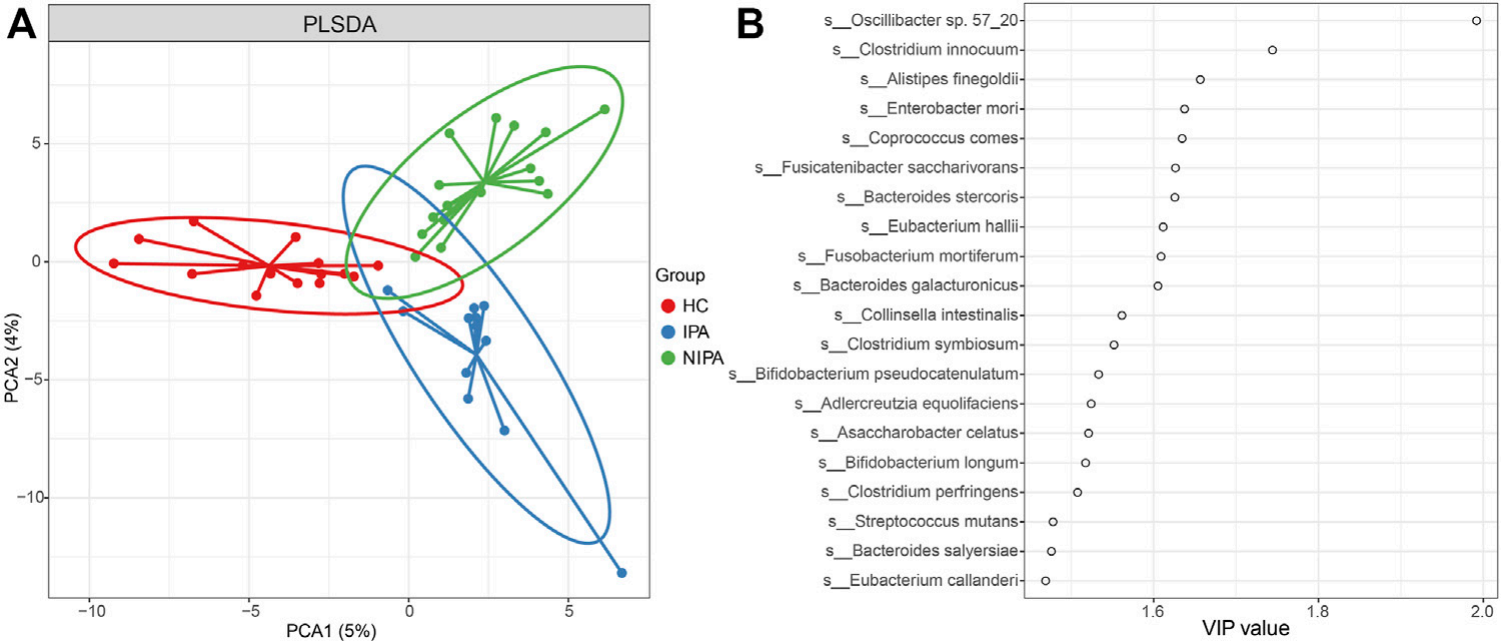
PLS-DA analysis of the gut microbiome. **(A)** PLS-DA score plot of species abundance samples from subjects with pituitary adenoma and healthy controls. **(B)** VIP score of PLS-DA.

We also performed partial least squares discriminant analysis (PLS-DA) to compare the gut microbial profiles. The results showed that there was a distinct clustering pattern between samples from subjects with pituitary adenoma and healthy controls (Figure 1A), thus reflecting their separations in microbial composition. The variable importance in projection (VIP) score for the gut microbiome showed that Oscillibacter sp. 57_20, and *Clostridium innocuum* was the top 2 microorganisms contributing to the group separation (Figure 1B).

### Differences in Specific Microbial Taxa Among the Three Groups

**FIGURE 2:**
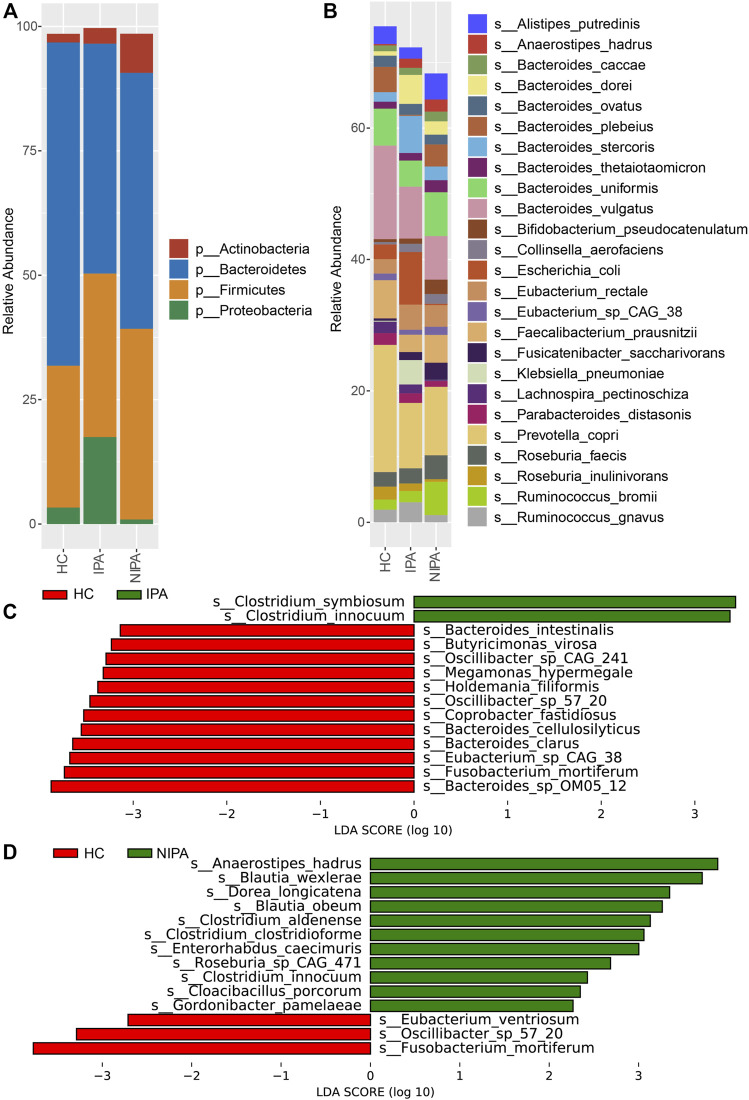
Microbial profiles of the gut microbiota in the PA and HC groups. **(A)** Relative abundances of the dominant phyla. **(B)** Relative abundances of the abundant species. **(C)** Differences in microbial species between the HC and IPA groups. **(D)** Different species between the HC and NIPA groups.

The average relative abundance of the dominant phyla shown in Figure 2A included Actinobacteria, Bacteroidetes, Firmicutes and Proteobacteria, where Bacteroidetes and Firmicutes accounted for more than 80%. Notably, the ratio of average relative abundance of Firmicutes/Bacteroidetes increased from HC group (0.44) to IPA (0.75) and NIPA (0.71) groups. Meanwhile, the predominant species were *Alistipes putredinis*, *Anaerostipes hadrus*, *Bacteroides caccae*, *Bacteroides dorei*, *Bacteroides ovatus*, *Bacteroides plebeius*, *Bacteroides stercoris*, *Bacteroides thetaiotaomicron*, *Bacteroides uniformis*, *Bacteroides vulgatus*, *Bifidobacterium pseudocatenulatum*, *Collinsella aerofaciens*, *Escherichia coli*, *Eubacterium rectale*, *Eubacterium* sp. CAG_38, *Faecalibacterium prausnitzii*, *Fusicatenibacter saccharivorans*, *Klebsiella pneumoniae*, *Lachnospira pectinoschiza*, *Parabacteroides distasonis*, *Prevotella copri*, *Roseburia faecis*, *Roseburia inulinivorans*, *Ruminococcus bromii* and *Ruminococcus gnavus* (Figure 2B). Compared to the HC group, the mean relative abundances of *Anaerostipes hadrus* (1.85% versus 0.21%, 1.39% versus 0.21%), *Bacteroides dorei* (2.02% versus 0.68%, 4.41% versus 0.68%), *Bacteroides stercoris* (2.08% versus 1.45%, 5.65% versus 1.45%), *Collinsella aerofaciens* (1.48% versus 0.37%, 1.27% versus 0.37%), *Eubacterium rectale* (3.29% versus 2.19%, 3.81% versus 2.19%), and *Fusicatenibacter saccharivorans* (2.62% versus 0.36%, 1.22% versus 0.36%) increased, whereas *Bacteroides vulgatus* (6.62% versus 14.24%, 7.91% versus 14.24%), *Faecalibacterium prausnitzii* (4.24% versus 5.80%, 2.65% versus 5.80%), and *Prevotella copri* (10.43% versus 19.36%, 9.95% versus 19.36%) decreased in both the NIPA and IPA groups.

Furthermore, we performed LEfSe analysis and revealed significantly different bacteria among the three groups. At the species level, specifically, the relative abundance of Clostridium innocuum was higher, while Oscillibacter sp. 57_20 and *Fusobacterium mortiferum* were lower in the NIPA group than in the HC group (Figure 2C). Likewise, enrichment of *Clostridium innocuum*, along with decreases of Oscillibacter sp. 57_20 and *Fusobacterium mortiferum* were also observed in the IPA group, compared to the HC group (Figure 2D). This result was consistent with the PLS-DA results.

**FIGURE 3:**
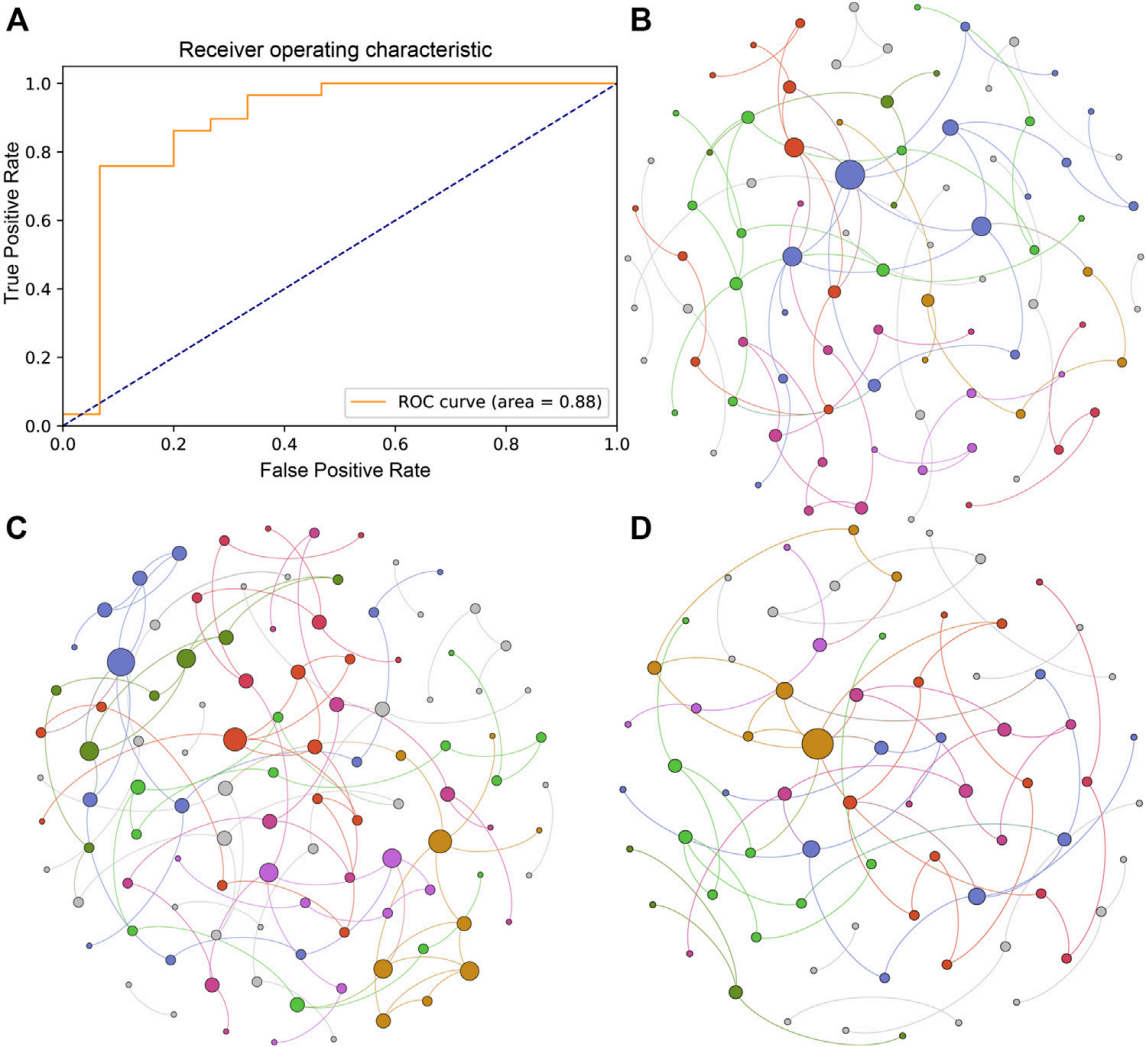
Classification mode and microbial ecological network of the PA and HC groups. **(A)** Receiver operating characteristic curve of the classification mode for pituitary adenoma using the SVM algorithm. **(B)** Microbial ecological network of the gut microbiome in the HC group. **(C)** Microbial ecological network of the gut microbiome in the IPA group. **(D)** Microbial ecological network of the gut microbiome in the NIPA group.

To explore the efficacy of pituitary adenoma classification based on the gut microbial profile, we used the SVM algorithm with 10-fold cross-validation to build a classification model at the species level. We identified 10 species that could be used to predict the occurrence of PA, including Oscillibacter sp. 57_20, *Fusobacterium mortiferum*, and *Clostridium innocuum*. The receiver operating characteristic curve of the model was shown in Figure 3A with an AUC of 0.88.

#### Different Microbial Ecological Networks in the Three Groups

Microorganisms in the gut inhibit, compete and cooperate with each other to form a stable ecological network. In this study, we used SpiecEasi software to investigate the microbial network at the genus level in the three groups separately. The results showed that there were 90 nodes and 84 edges in the microbial ecological network of the HC group (Figure 3B). Compared to the HC group, a more complex microbial network was present in the IPA group, with 108 nodes and 113 edges (Figure 3C), whereas a simpler network was observed in the NIPA group, with 72 nodes and 70 edges (Figure 3D). The results showed alterations in the gut microbiota in subjects with pituitary adenoma, and the change patterns were different between the IPA and NIPA groups.

### Functional Alterations Between the PA and HC Groups

**FIGURE 4:**
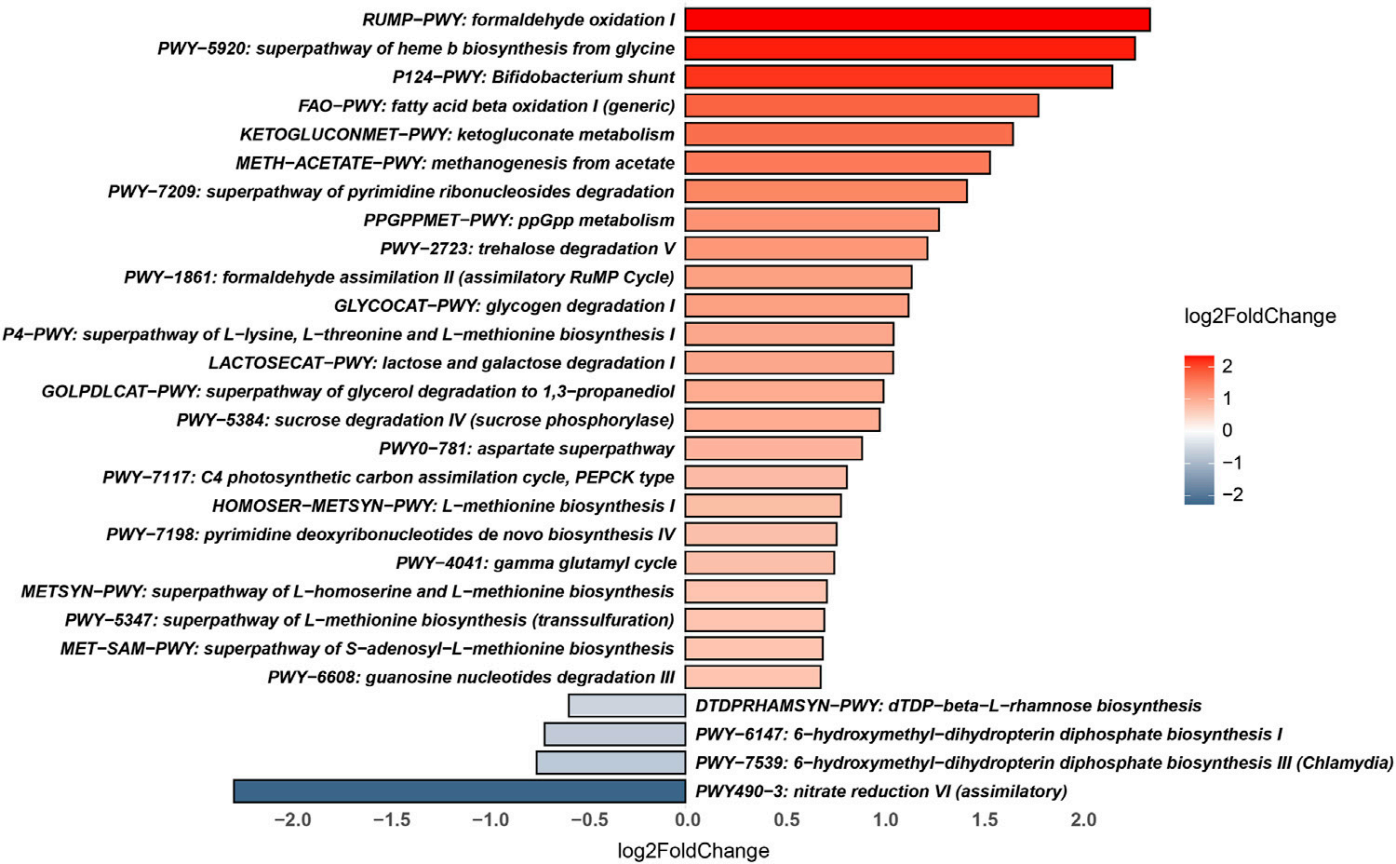
Significantly different metabolic pathways between the PA and HC groups.

The metabolic pathways of the PA and HC groups were predicted using HUMAnN3 software against the MetaCyc database. The pathways that were significantly enriched in the PA group were “formaldehyde oxidation I,” “superpathway of haem b biosynthesis from glycine,” “Bifidobacterium shunt,” “fatty acid beta-oxidation I,” “ketogluconate metabolism,” “methanogenesis from acetate,” “superpathway of pyrimidine ribonucleoside degradation,” “ppGpp metabolism,” “trehalose degradation V,” “formaldehyde assimilation II,” “glycogen degradation I,” “superpathway of L-lysine, L-threonine and L-methionine biosynthesis I,” “lactose and galactose degradation I,” “superpathway of glycerol degradation to 1,3-propanediol,” “sucrose degradation IV,” “aspartate superpathway,” “C4 photosynthetic carbon assimilation cycle,” “L-methionine biosynthesis I,” “pyrimidine deoxyribonucleotides *de novo* biosynthesis IV,” “gamma glutamyl cycle,” “superpathway of L-homoserine and L-methionine biosynthesis,” “superpathway of S-adenosyl-L-methionine biosynthesis,” and “guanosine nucleotides degradation III.” However, the microbial functions related to “dDTP-beta-L-rhamnose biosynthesis,” “6-hydroxymethyl-dihydropterin diphosphate biosynthesis I and III,” and “nitrate reduction VI” were higher in the gut microbiome of the HC group (Figure 4).

## Discussion

Pituitary adenoma (PA) is the third most common central nervous system tumour among adults, and it arises in the anterior pituitary gland [5]. It is linked to serious morbidity because of dysregulated pituitary hormone secretion [5]. Most PAs are benign, and approximately 35% exhibit invasive behaviours [3]. Accumulating evidence suggests that dysbiosis of the gut microbiome plays an important role in the central nervous, endocrine and neuroendocrine systems [8, 10, 11]. However, little attention has been given to the examination of gut microbial characteristics in PA.

In this study, we divided PA patients into IPA and NIPA subgroups and utilized high-throughput metagenomic sequencing technology to identify the differences in the microbial community in the IPA, NIPA, and HC groups. The results demonstrated that the microbial community separated well between the PA and HC groups using PLS-DA analysis, where Oscillibacter sp. 57_20 and *Clostridium innocuum* contributed substantially, and different microbial ecological networks of the three groups were observed. The ratio of Firmicutes/Bacteroidetes increased in both IPA and NIPA groups, compared to HC group. Furthermore, we observed that several bacterial taxa were differentially enriched using LEfSe analysis. At the species level, *Clostridium innocuum* was significantly overrepresented in both the IPA and NIPA groups compared with the HC group, whereas Oscillibacter sp. 57_20 and *Fusobacterium mortiferum* were enriched in the HC group. Based on these different species, a good classification effect of PA identification was observed using the SVM algorithm with an AUC of 0.88. The abovementioned species might serve as common contributors for discriminating individuals with a high risk of PA.

No matter animals and humans, the obese subjects all exhibit higher Firmicutes/Bacteroidetes ratios, compared with normal weight subjects [22]. Consistent with the previous result, a higher Firmicutes/Bacteroidetes ratio was also found in PA group in this study. These might indicate that high Firmicutes/Bacteroidetes ratio was related to endocrine system, which should be confirmed with more datasets. Previous research suggests that *Clostridium innocuum* induced inflammation, oedema and necrosis in a mouse ileal loop mode [23]. Additionally, *C. innocuum* has been reported to be the second most common Clostridial species causing extraintestinal infections in Taiwan, and a variety of infections have also been observed in other populations [23]. In another study, *C. innocuum* was implicated in tissue remodelling and inflammation in Crohn’s disease [24]. However, the role of *C. innocuum* in PA remains unknown. On the other hand, Oscillibacter sp. 57_20 was found to associate with decreased BMI [25]. Notably, a significant decrease in the abundance of the genus Oscillibacter was observed in newly diagnosed acromegaly patients with adenoma in the pituitary gland [13]. It was also found that the abundance of Oscillibacter sp. 57_20 was associated with plasma cysteine levels [26] and some microRNA levels [27]. The role of Oscillibacter sp. 57_20 in pituitary tumorigenesis needs to be defined with experimental and animal studies in the future. Furthermore, *Fusobacterium mortiferum* significantly increased in APC gene mutation patients with intestinal adenomatous polyps [28, 29]. Nonetheless, F. mortiferum is able to produce bacteriocin-like substances with an inhibitory effect on a number of species. Therefore, it is difficult to know whether the decreased abundance of *F. mortiferum* in PA patients is due to its potential pathogenicity or defence mechanism, and more studies are needed to determine this possibility.

Fatty acids are associated with the hypothalamic–pituitary–adrenal axis [30] and play an important role in the regulation of growth hormone concentrations [31]. In addition, exposure to low concentrations of formaldehyde is helpful to induce hypothalamic–pituitary–adrenal activity, such as corticotropin releasing hormone and the adrenocorticotropin hormone [32]. In this study, the metabolic pathways “formaldehyde oxidation I” and “fatty acid beta-oxidation I” were significantly altered in PA patients. It is well known that short-chain fatty acids (SCFAs) produced by microbiota can modulate immunity, increase growth hormone activity and insulin sensitivity, and regulate the neuroendocrine system [10, 33, 34]. A decreasing pattern of the SCFA producer Faecalibacterium prausnitzii was observed in both the IPA and NIPA groups compared to the HC groups. This might be associated with the pathogenesis of PA. Furthermore, many pathways of glucose metabolism (“glycogen degradation I,” “lactose and galactose degradation I” and “sucrose degradation IV”), which are related to the endocrine system, were significantly changed in the PA patients. Overall, our findings add evidence that PA patients have an altered gut microbiome. However, larger prospective studies should be conducted to investigate the underlying mechanism.

Despite the above findings, the main limitations of our study were the small sample size, and all the participants were recruited from a single geographic area without consideration of possible confounding factors, such as diet, culture, and demographics. Enlargement of the sample size from different regions and animal experiments are needed to further characterize the alterations of gut microbiota in PA patients.

In conclusion, we identified dysbiosis of the gut microbiome in PA patients. The aberrant gut microbiota of PA patients might serve as potential biomarkers for early risk detection. These findings could help improve the understanding of pituitary adenoma aetiology and support the development of new treatment options based on modulation of the gut microbiome.

## Data Availability

The raw sequencing data were deposited in the Sequence Read Archive under the accession number of PRJNA799832.
